# Exclusive breastfeeding lowers the odds of childhood diarrhea and other medical conditions: evidence from the 2016 Ethiopian demographic and health survey

**DOI:** 10.1186/s13052-021-01115-3

**Published:** 2021-08-03

**Authors:** Tesfahun Mulatu, Nigus Bililign Yimer, Birhan Alemnew, Melese Linger, Misgan Legesse Liben

**Affiliations:** 1 School of Public Health, College of Health Science, Woldia University, P.o.box: 400, Woldia University, Weldiya, Amhara Ethiopia; 2 School of Midwifery, College of Health Science, Woldia University, Weldiya, Amhara Ethiopia; 3 Department of Medical Laboratory Science, College of Health Science, Woldia University, Weldiya, Amhara Ethiopia

**Keywords:** Cough, Breastfeeding, Diarrhea, Fever, Sub-Saharan Africa

## Abstract

**Background:**

Lack of exclusive breastfeeding during the first 6 months of infant life contributes to childhood morbidity and mortality. This study aimed to investigate the association of exclusive breastfeeding and childhood illnesses in Ethiopia.

**Methods:**

A secondary data analysis was conducted using data from the 2016 Ethiopian Demographic and Health Survey (EDHS). Descriptive and multivariable logistic regression analyses were carried out.

**Results:**

A total of 1034 mother-infant pairs were included in the analysis. The overall magnitude of exclusive breastfeeding among infants aged under 6 months was 87.6% (95% CI: 84.3–90.3%). Compared to infants who were non-exclusively breastfed, the odds of having an illness with fever in the last 2 weeks among infants who were exclusively breastfed decreased by 66% (AOR: 0.34; 95% CI: 0.16, 0.75). Similarly, exclusively breastfed infants had lower odds of having an illness with a cough (AOR: 0.38; CI: 0.20, 0.72) and having diarrhea (AOR: 0.33; CI: 0.13, 0.83) compared to non-exclusively breastfed infants.

**Conclusion:**

Exclusive breastfeeding lowers the odds of an illness with fever, illness with cough and diarrhea. The findings of this study implicate the need for promotion of exclusive breastfeeding in the country.

**Supplementary Information:**

The online version contains supplementary material available at 10.1186/s13052-021-01115-3.

## Background

Breast milk provides ideal nutrition to meet the infant’s needs for growth and development. It protects against many infections, and may prevent some infant deaths. Hence, breastfeeding has well established short and long-term benefits in childhood. It reduces morbidity, mortality and the risk of hospitalization from diarrhea and respiratory tract infections [1].

Exclusive breastfeeding is an infant’s consumption of only breast milk in the first 6 months of life. The World Health Organization (WHO) recommend that 95% of children younger than 1 month and 90% of those younger than 6 months should be exclusively breastfed, and 90% of those aged 6–23 months should be partly breastfed. However, in low and middle-income countries, only 37% of children younger than 6 months of age are exclusively breastfed [2]. In Ethiopia, about 77% of infants scored low and medium breastfeeding performance index during the first 5 months of life [3], and only 58% of infants under age 6 months are exclusively breastfed [4].

Breastfeeding provides all the nutrients and water that a baby needs to grow and develop in the first 6 months [5, 6]. Exclusive breastfeeding has well-established short-term and long term benefits, particularly in the reduction of childhood morbidity and mortality due to diarrhea and respiratory infection [7]. Lower breastfeeding index has been associated with increased risks of diarrhea and fever [3]. Furthermore, an estimated 1.3 million lives would be saved worldwide by promoting exclusive breastfeeding [1].

The government of Ethiopia has adapted infant and young child feeding guidelines since 2004 [8] and the national nutrition program in 2013 [9] to unlock the lifesaving potential of optimal breastfeeding practices. The guideline is based on WHO recommendations that give emphasis on exclusive breastfeeding. Furthermore, the health extension program in Ethiopia aims at improving proper infant and young child nutrition, for instance, promotion of exclusive breastfeeding [8].

Nongovernmental organizations are also addressing the issue of exclusive breastfeeding in different regions of the country through advocacy, community mobilization, and mass communication [9]. However, little is known about the impact of different breastfeeding practices on childhood illnesses in Ethiopia. Therefore, this study aimed to investigate the association of exclusive breastfeeding and childhood illnesses in Ethiopia.

## Methods

### Study setting and design

The 2016 EDHS was designed to provide up-to-date estimates of key demographic and health indicators in Ethiopia. A detailed description of the study design and methodology of the 2016 is found elsewhere [8]. In brief, a stratified two-stage random sampling design was used to collect data from a nationally representative sample. In the first stage, a total of 645 Enumeration Areas (EA) (202 in urban areas and 443 in rural areas) were selected with probability proportional to EA size and with independent selection in each sampling stratum. In the second stage, a fixed number of 28 households per cluster were selected with an equal probability systematic selection from the newly created household listing. A total of 18,008 households were selected, of which 17,067 were occupied and 16,650 were successfully interviewed, thus yielding a household response rate of 98%. In the selected households, the response rate among women age 15–49 was 94.6%. The sampling frame for the current study consisted of 1036 infants last-born infants aged less than 6 months, currently living with their mothers. We excluded women with missing data on the question related to the outcomes of interest, exclusive breastfeeding practices and other covariates adjusted in the multivariable model. The final analytic sample consisted of 1034 participants.

### Outcome of interest

The outcome of interest was childhood illnesses. Three childhood illnesses namely: illness with fever, diarrhea and illness with cough were assessed by asking the mother to recall if the infant had any of these illnesses in the last 2 weeks preceding the survey. Each of these variables was dummy coded to reflect the presence or absence of these illnesses. Women who responded “Don’t know” were excluded.

### Exposure assessment

Exclusive breastfeeding was defined based on mother’s recall on feeds given to the infant on the previous day. Specifically, mothers were asked whether or not infants received any solid, semi-solid or soft foods 24 h preceding the survey.

### Covariates

Based on existing literature, the following covariates were selected: age of mother at infant’s birth, household wealth index, educational level, place of delivery, place of residence, region of residence, antenatal care (ANC checkup), mode of delivery, age of infant, sex of infant, birth order, vaccination status, source of drinking water, wealth index and toilet facility.

### Statistical analysis

Data were analyzed using SPSS version 23 (IBM Corp. Released 2015. IBM SPSS Statistics for Windows, Version 23.0. Armonk, NY: IBM Corp.). Descriptive statistics and cross-tabulation were performed to describe the study variables. Bivariate associations between each covariate and exclusive breastfeeding were first examined using Rao-Scott chi-square test. Univariable and multivariable logistic regression models were employed to determine the association between exclusive breastfeeding and childhood illness. Variance inflation factor (VIF) was used to assess the presence of collinearity. Using a conservative threshold VIF value of 4, no co-linearity was detected. Crude odds ratios (COR) and adjusted odds ratios (AOR) were presented with 95% confidence intervals. Each covariate were included in the multivariable model regardless of their statistical significance in the univariable analysis. In the final multivariable logistic regression model, the association of EBF with childhood illness was declared statistically significant at *p* value < 0.05. As recommended by the DHS Program, sample weights that account for complex survey design and unequal probabilities of selection were incorporated in all analyses [10].

### Ethical issues

The data were downloaded once approval was obtained from Measure DHS. The original DHS data were collected in confirmation with international and national ethical guidelines. The 2016 EDHS protocol was reviewed and approved by the Federal Democratic Republic of Ethiopia Ministry of Science and Technology and the Institutional Review Board of ICF International.

## Results

Table 1 displays characteristics of the study sample. Overall, the prevalence of exclusive breastfeeding among infants aged under 6 months was 87.6% (95% CI: 84.3–90.3%). The prevalence of illness with cough in under-6 month infants was 17.9% (95% CI: 14.7–21.7%). About 12% of infants (11.8%; 95% CI: 9.4–14.8%) had illness with fever and 7.4% (95% CI: 5.4–10.1%) had diarrhea in the last 2 weeks. The mean (±SE) age of the mothers and their under six infants were 27.57 (0.31) years and 2.50 (0.08) months respectively. Majority (71.5%) of the mothers were in the age group of 20 to 34 years. About 8 out of 9 mothers (88.7%) were rural residents, and majority of them (59.4%) had no formal education. Women who were in the poorest and poor wealth index were more likely to exclusively breastfeed (Table 1).

**Table 1:** Characteristics of the study sample by exclusive breastfeeding status in Ethiopia, EDHS 2016 (*n* = 1034)

Variables		Exclusive BF		
	Overall	No	Yes	
	n (wt.%)	n (wt.%)	n (wt.%)	***p***
**Maternal age**				
< 20	143 (13.3)	18 (9.4)	125 (13.9)	0.327
20–34	753 (71.5)	75 (69.3)	678 (71.8)	
35+	138 (15.2)	17 (21.3)	121 (14.4)	
**Wealth Index**				
Poorest	394 (24.4)	39 (17.5)	355 (25.4)	0.057
Poorer	164 (23.4)	20 (25.6)	144 (23.1)	
Middle	123 (18.8)	17 (25.2)	106 (17.9)	
Richer	138 (18.1)	19 (26.4)	119 (17.0)	
Richest	215 (15.2)	15 (5.2)	200 (16.6)	
**Place of residence**				
Urban	203 (11.3)	16 (7.7)	187 (11.8)	0.265
Rural	831 (88.7)	94 (92.3)	737 (88.2)	
**Education level of mothers**				
No education	606 (59.4)	73 (63.6)	533 (58.8)	0.797
Primary	288 (30.4)	27 (25.2)	261 (31.2)	
Secondary	103 (8.0)	7 (9.4)	96 (7.8)	
Higher	37 (2.2)	3 (1.8)	34 (2.3)	
**Birth order**				
1	216 (22.1)	21 (16.5)	195 (22.9)	0.098
2–3	318 (28.2)	38 (25.5)	280 (28.6)	
4–6	328 (30.9)	27 (27.0)	301 (31.5)	
≥ 7	172 (18.7)	24 (30.9)	148 (17.0)	

*Abbreviations: Wt: weighted. BF: Breastfeeding*

Only 8.8% of the households included in this analysis had improved toilet facility and nearly half of the households (54.1%) had improved drinking water source. From those who were non- exclusively breastfed about 87% of them had unimproved toilet facility or had no toilet facility (open defecation) at all. One third of the mothers (31.3%) had attended 4 or more ANC visits. Half of the mothers with non-exclusively breastfed neonates had no ANC visit and three forth of them had delivered at home which was significantly different (*p* value 0.037) from those mothers with exclusively breastfed neonates. Nearly all of the mothers had vaginal delivery (97%). Majority (73.0%) of the mothers initiated breastfeeding within 1 h of birth and about 7 out of 10 mothers had given anything other than breast milk. About 9 out of 10 (91.4%) under six infants were bottle-fed and there was significant difference (*p* value < 0.001) in bottle feeding status between exclusively and non-exclusively breastfed neonates (Table 2).

**Table 2:** Characteristics of mother-infant pairs in Ethiopia, EDHS 2016 (n = 1034)

Variables		Exclusive BF		***p***
	Overall	No	Yes	
	n (%)	n (wt.%)	n (wt.%)	
**Sex of infant**				
Male	512 (49.2)	59 (54.7)	453 (48.4)	0.356
Female	522 (50.8)	51 (45.3)	471 (51.6)	
**Ever vaccinated**				
No	445 (49.6)	39 (40.2)	406 (50.9)	0.114
Yes	589 (50.4)	71 (59.8)	518 (49.1)	
**Toilet facility**				
Open defecation	459 (38.0)	51 (31.8)	408 (38.8)	0.556
Unimproved facility	401 (53.2)	45 (56.1)	356 (52.8)	
Improved facility	174 (8.8)	14 (12.1)	160 (8.4)	
**Drinking water source**				
Improved	624 (54.1)	60 (49.7)	564 (54.7)	0.476
Unimproved	410 (45.9)	50 (50.3)	360 (45.3)	
**ANC visit**				
No visit	336 (34.5)	52 (49.3)	284 (32.4)	0.049
1–3 visits	341 (34.2)	28 (22.2)	313 (35.9)	
≥ 4 visits	357 (31.3)	30 (28.5)	327 (31.7)	
**Place of delivery**				
Home	604 (63.4)	80 (75.7)	524 (61.7)	0.037
Health institution	430 (36.6)	30 (24.3)	400 (38.3)	
**Mode of delivery**				
Vaginal	996(97.0)	103 (95.4)	893 (97.3)	0.368
Cesarean	38 (3.0)	7 (4.6)	31 (2.7)	
**Currently breastfeeding**				
No	45 (4.7)	5 (7.0)	40 (4.4)	0.383
Yes	989 (95.3)	105 (93.0)	884 (95.6)	
**Time of initiation of breastfeeding**				
≤ 1 h	716 (73.0)	69 (70.7)	647 (73.4)	0.715
> 1 h	304 (27.0)	39 (29.3)	265 (26.6)	
**Given anything other than breast milk**				
No	858 (93.1)	76 (88.3)	782 (93.8)	0.068
Yes	162 (6.9)	32 (11.7)	130 (6.2)	
**Bottle feeding**				
No	920 (91.4)	86 (76.9)	834 (93.4)	< 0.001
Yes	114 (8.6)	24 (23.1)	90 (6.6)	

Abbreviations: *BF* Breastfeeding; *ANC* Antenatal care

Table 3 presents results from the univariable and multivariable logistic regression analyses. Exclusive breastfeeding was significantly associated with illness with fever, illness with cough and diarrhea. The odds of developing illness with fever in those infants exclusively breastfed were by 66% (AOR: 0.34 (CI: 0.16, 0.75)) lower than those who did not fed exclusively. Those infants who were exclusively breastfeeding had lower odds of developing cough and diarrhea by 62% (AOR: 0.38 (CI: 0.20, 0.72)) and 67% (AOR: 0.33 (CI: 0.13, 0.83)) respectively as compared to those who did not fed exclusively (Table 3).

**Table 3:** Association between exclusive breastfeeding and diarrhea, illness with fever and illness with cough among infants aged less than 6 months in Ethiopia, EDHS 2016

	Illness with Fever in the last 2 weeks				Illness with Cough in the last 2 weeks				Diarrhea in the last 2 weeks			
	Yes	No	COR (95% CI)	AOR^x^ (95% CI)	Yes	No	COR (95% CI)	AOR^y^(95% CI)	Yes	No	COR (95% CI)	AOR^z^ (95% CI)
**EBF**												
Yes	92	832	0.30 (0.15, 0.59)*	0.34 (0.16, 0.75)*	148	776	0.38 (0.20, 0.72)*	0.38 (0.20 0.72)*	54	870	0.23 (0.11, 0.48)*	0.33 (0.13, 0.83)*
No	30	80	1.00	1.00	37	73	1.00	1.00	23	87	1.00	1.00

*Statistically significant at *P* < 0.05. EBF: Excusive Breastfeeding: *COR* Crude Odds Ratio; AOR: Adjusted Odds Ratio CI: Confidence Interval

^x^Adjusted for wealth index, birth order, age at birth, residence, ANC visit, place of delivery, mode of delivery, vaccination, source of drinking water, infant sex and infant age

^y^Adjusted for wealth index, birth order, age at birth, residence, ANC visit, education level, place of delivery, mode of delivery, vaccination, infant sex, source of drinking water and infant age

^z^Adjusted for wealth index, birth order, residence, vaccination, source of drinking water, type of toilet facility and infant age

## Discussion

In the current study, the overall magnitude of exclusive breastfeeding among infants aged less than 6 months was 87.6% and exclusive breastfeeding (EBF) was significantly associated with decreased odds of childhood illnesses. EBF lowered the odds of illness with fever, illness with cough and diarrhea in the last 2 weeks. These associations were independent of potential confounders. Our findings adds to the extant literature by being the first to examine the association between exclusive breastfeeding and childhood illnesses in Ethiopia.

The present study showed that exclusive breastfeeding decreased the odds of illness with fever by 66%. Exclusive breastfeeding is a strong predictor for infant survival in Ethiopia [11]. Evidence from a Scottish birth cohort reported formula-fed infants had increased rate of hospitalization for gastrointestinal and respiratory tract infections and fevers [12]. A randomized controlled trial revealed that not breastfeeding was associated with increased risks for childhood morbidities including fever, and gastroenteritis [13]. Additionally, longer duration of breastfeeding complemented with antimalarial drugs was linked with decreased childhood mortality [14]. Moreover, longer breastfeeding duration decreased the odds of recurrent cough [15]. Discontinuation of breastfeeding when the baby had fever/cold has been reported elsewhere [16]. Inadequate nutritionals requirement in early life can result in reduced protection against infections [17]. Because of the greater total immunoglobulin concentrations [18], longer duration and frequent breastfeeding might have important protective effect for infections.

The current study revealed there was a 62% lower odds of developing cough among exclusively breastfed infants. This finding is consistent with findings from other studies. A longitudinal study conducted in Assam, India revealed low birth weight babies without exclusive breastfeeding were at higher risk for morbidities like acute respiratory tract infections [19]. Similarly, a case-control study in Italy revealed that upper and lower respiratory tract infections were strongly associated with lack of breastfeeding [20]. Indeed, lack of breastfeeding was associated with lower respiratory tract infections in a randomized controlled trial [13]. Exclusive breastfeeding decreased the odds of rhinitis in Amazon islands, Brazil [21]. An ordinal increase in breastfeeding duration was associated with decreased risk of recurrent cough in adult life [22]. Moreover, a cohort study in Vietnam revealed exclusive breastfeeding was associated with decreased odds of admission for pneumonia [23]. Similarly, an eight-country cohort finding showed the protective effect of exclusive breastfeeding against respiratory tract infections [24]. In Indonesia, delayed initiation of breastfeeding was associated with increased risks of cough and difficulty in breathing during the first 6 months of life [25]. On the other hand, breastfeeding was not a protective factor for pertussis among unvaccinated infants in Italy [26]. This might be due to the baseline immune capacity of the infants or the nature of the disease against immunologic content of the breast milk. Stakeholders need to design appropriate interventions to help mothers breastfeed their babies in case of childhood illnesses.

In this study, exclusive breastfeeding decreased the odds of diarrhea by 67%. This finding is in line with a study conducted in rural Nepal, where suboptimal breastfeeding practices were associated with higher odds of childhood diarrhea [27]. Early life diarrheal disease among non-breastfed infants was associated with shorten telomere length in adult immune cells [28]. A cross-sectional study conducted in north Gondar zone and Afar region of Ethiopia reported children without breastfeeding/not-exclusive breastfeeding practices were at higher risk for diarrheal diseases [29, 30]. A Demographic and Health Survey (DHS) analysis in nine Sub-Saharan African countries revealed exclusive breastfeeding was significantly associated with lower risk of diarrhea [31]. A similar study using the DHS of Tanzania reported the protective effect of exclusive breastfeeding against diarrheal diseases [32]. Additionally, a cohort study in eight countries revealed the protective effect of exclusive breastfeeding on episodes of diarrhea [24]. Prenatal anxiety which might be related to less exclusive breastfeeding [33] and myths about discontinuation of breastfeeding during diarrheal episodes in some communities [34] should be discouraged [20]. As mothers are more receptive for skill-based interactive demonstrations targeted to exclusive breastfeeding [35], designing novel strategies tailored to exclusive breastfeeding is needed.

This study is not without limitations. First, the cross-sectional nature of the study precludes drawing conclusions about the influence of exclusive breastfeeding on childhood illnesses. Second, exclusive breastfeeding and childhood illnesses may be prone to self-reported, recall and social desirability biases. Third, although 24-h recall has been widely used to measure infant feeding practices, a recent study in Ethiopia have shown that a single 24-h recall overestimates exclusive breastfeeding practices [36]. Additionally, this study is prone to selection bias, misclassification and residual confounding. The relatively large sample size, availability of detailed data on confounders, and standardized instruments, high-quality data collection are some of the strengths of the current study.

## Conclusion

Exclusive breastfeeding was significantly associated with decreased the odds of illness with fever, illness with cough and diarrhea in a representative, sample of Ethiopian infants. This association remained independent of age of mother at infant’s birth, household wealth index, educational level, place of delivery, place of residence, region of residence, antenatal care (ANC checkup), mode of delivery, age of infant, sex of infant, birth order, vaccination status, source of drinking water, wealth index and type of toilet facility. Promoting exclusive breastfeeding throughout the country would have a greater contribution for infant health and development. Hence, in developing countries including Ethiopia, interventions targeting women who had no history of antenatal checkup and home delivery are the most cost effective intervention to strengthen exclusive breastfeeding.

## Supplementary Information

**Additional file 1.**

## Data Availability

The datasets used for this manuscript are available from the corresponding author upon reasonable request.

## Abbreviations

ANC: Antenatal Care
AOR: Adjusted Odds ratio
COR: Crude Odds Ratio
DHS: Demographic and Health Survey
EA: Enumeration areas
EBF: Exclusive Breastfeeding
SE: Standard error
VIF: Variance inflation factor
WHO: World Health Organization
