# Supplementary material for: Exclusive breastfeeding lowers the odds of childhood diarrhea and other medical conditions: evidence from the 2016 Ethiopian demographic and health survey
Source: Ital J Pediatr. 2021 Aug 3;47:166. doi: 10.1186/s13052-021-01115-3 (PMC8335997; doi:10.1186/s13052-021-01115-3)
Supplement: Supplementary file 1 — Additional file 1. [file 13052_2021_1115_MOESM1_ESM.pdf]

NO.	QUESTIONS AND FILTERS	LAST BIRTH<br>NAME ________	NEXT-TO-LAST BIRTH<br>NAME ________
609	CHECK 464: EVER BREASTFED?<br>YES ☐ → a) Now I would like to know how much (NAME) was given to drink during the diarrhea including breastmilk. Was (NAME) given less than usual to drink, about the same amount, or more than usual to drink?<br>IF LESS, PROBE: Was (NAME) given much less than usual to drink or somewhat less?<br>NO ☐ → b) Now I would like to know how much (NAME) was given to drink during the diarrhea. Was (NAME) given less than usual to drink, about the same amount, or more than usual to drink?<br>IF LESS, PROBE: Was (NAME) given much less than usual to drink or somewhat less?	MUCH LESS . . . . 1<br>SOMEWHAT LESS . . . . 2<br>ABOUT THE SAME . . . . 3<br>MORE . . . . 4<br>NOTHING TO DRINK . . . . 5<br>DON'T KNOW 8	MUCH LESS . . . . 1<br>SOMEWHAT LESS . . . . 2<br>ABOUT THE SAME . . . . 3<br>MORE . . . . 4<br>NOTHING TO DRINK . . . . 5<br>DON'T KNOW 8
610	When (NAME) had diarrhea, was (NAME) given less than usual to eat, about the same amount, more than usual, or nothing to eat?<br>IF LESS, PROBE: Was (NAME) given much less than usual to eat or somewhat less?	MUCH LESS . . . . 1<br>SOMEWHAT LESS . . . . 2<br>ABOUT THE SAME . . . . 3<br>MORE . . . . 4<br>STOPPED FOOD . . . . 5<br>NEVER GAVE FOOD . . . . 6<br>DON'T KNOW 8	MUCH LESS . . . . 1<br>SOMEWHAT LESS . . . . 2<br>ABOUT THE SAME . . . . 3<br>MORE . . . . 4<br>STOPPED FOOD . . . . 5<br>NEVER GAVE FOOD . . . . 6<br>DON'T KNOW 8
611	Did you seek advice or treatment for the diarrhea from any source?	YES . . . . 1<br>NO . . . . 2<br>(SKIP TO 615) ←	YES . . . . 1<br>NO . . . . 2<br>(SKIP TO 615) ←

NO.	QUESTIONS AND FILTERS	LAST BIRTH<br>NAME ________	NEXT-TO-LAST BIRTH<br>NAME ________
615	Was (NAME) given any of the following at any time since (NAME) started having the diarrhea:<br>a) A fluid made from a special packet called LEMLEM?<br>b) A government-recommended homemade fluid?<br>c) Zinc tablets or syrup?	YES NO DK<br>a) FLUID FROM ORS PACKET . . 1 2 8<br>b) HOMEMADE FLUID . . . . . 1 2 8<br>c) ZINC 1 2 8	YES NO DK<br>a) FLUID FROM ORS PACKET . . 1 2 8<br>b) HOMEMADE FLUID . . . . . 1 2 8<br>c) ZINC 1 2 8
616	CHECK 615:<br>ANY 'YES' ⬜ / ALL 'NO' OR 'DK' ⬜<br>a) Was anything else given to treat the diarrhea? / b) Was anything given to treat the diarrhea?	YES . . . . 1<br>NO . . . . 2<br>(SKIP TO 618)<br>DON'T KNOW 8	YES . . . . 1<br>NO . . . . 2<br>(SKIP TO 618)<br>DON'T KNOW 8
617	CHECK 615:<br>ANY 'YES' ⬜ / ALL 'NO' OR 'DK' ⬜<br>a) What else was given to treat the diarrhea? / b) What was given to treat the diarrhea?<br>Anything else? / Anything else?<br>RECORD ALL TREATMENTS GIVEN.	**PILL OR SYRUP**<br>ANTIBIOTIC . . . . A<br>ANTIMOTILITY . . . . B<br>OTHER (NOT ANTIBIOTIC OR ANTIMOTILITY) . . . . . C<br>UNKNOWN PILL OR SYRUP . . . . D<br>**INJECTION**<br>ANTIBIOTIC . . . . E<br>NON-ANTIBIOTIC . . . . F<br>UNKNOWN INJECTION . . . . G<br>(IV) INTRAVENOUS . . . . H<br>HOME REMEDY/ HERBAL MEDICINE . . . . I<br>OTHER ________ X<br>(SPECIFY)	**PILL OR SYRUP**<br>ANTIBIOTIC . . . . A<br>ANTIMOTILITY . . . . B<br>OTHER (NOT ANTIBIOTIC OR ANTIMOTILITY) . . . . . C<br>UNKNOWN PILL OR SYRUP . . . . D<br>**INJECTION**<br>ANTIBIOTIC . . . . E<br>NON-ANTIBIOTIC . . . . F<br>UNKNOWN INJECTION . . . . G<br>(IV) INTRAVENOUS . . . . H<br>HOME REMEDY/ HERBAL MEDICINE . . . . I<br>OTHER ________ X<br>(SPECIFY)
618	Has (NAME) been ill with a fever at any time in the last 2 weeks?	YES . . . . 1<br>NO . . . . 2<br>DON'T KNOW 8	YES . . . . 1<br>NO . . . . 2<br>DON'T KNOW 8
620	Has (NAME) had an illness with a cough at any time in the last 2 weeks?	YES . . . . 1<br>NO . . . . 2<br>DON'T KNOW 8	YES . . . . 1<br>NO . . . . 2<br>DON'T KNOW 8
621	Has (NAME) had fast, short, rapid breaths or difficulty breathing at any time in the last 2 weeks?	YES . . . . 1<br>NO . . . . 2<br>(SKIP TO 623)<br>DON'T KNOW 8	YES . . . . 1<br>NO . . . . 2<br>(SKIP TO 623)<br>DON'T KNOW 8
